# Laparoscopic Cholecystectomy in the elderly

**DOI:** 10.1186/1471-2318-11-S1-A59

**Published:** 2011-08-24

**Authors:** S Spiezia, S Grassia, D De Rosa, DG Palmieri, N Carlomagno, C Dodaro, A Renda

**Affiliations:** 1 University Department of Surgical, Anesthesiology-Reanimation and Emergency Sciences. University of Naples "Federico II", Italy

## Background

Laparoscopic cholecystectomy (LC) has shown benefits, in order to become the universal gold standard for cholelithiasis and other diseases of the gallbladder. Such pathologies are very common in the elderly, but in these cases LC might pose problems because of the comorbidity frequently associated. The aim of this study is to evaluate the outcome of LC in the elderly.

## Materials and methods

A retrospective study was conducted on 204 patients affected by symptomatic cholelithiasis and other diseases of the gallbladder observed during the last four years (from January 2006 to December 2009). Patients were divided into two groups according to their age :a) < 75 y. o. – b) >= 75 y. o., to compare operative time, conversion rate to open cholecystectomy, complication rate and length of stay. Other parameters were evaluated such as sex, comorbidity (cardiovascular and respiratory diseases, hypertension, diabetes), previous abdominal surgery, diagnosis of acute cholecystitis and lithiasis of the common bile duct, biochemistry (leukocytosis, hyperbilirubinemia, transaminase) and ultrasonography.

## Results

There were 144 patients aged < 75 y. o. (Group a) and 60 patients > 75 y. o. (Group b). The majority of the patients in each group were female (90 in Group a and 40 in group b). 184 patients underwent LC and the remaining 20 underwent open surgery. Comorbidity was higher in the elderly patients ( 40% vs 22%).

Open cholecystectomies were more frequently performed in the elderly (13.33% vs 8.3%) because of previous surgery, cardiac and/or respiratory failure and gangrenous cholecystis. Scleroatrophy and acute inflammation were the main causes of conversion and had a marginal independent effect on the development of complications among elderly patients. After open cholecystectomy there were no complications but the hospital stay was longer Table 1.

**Table 1:** 

	Group a	Group b
**Conversion rate**	2.27 %	1.92 %
**Complication rate**	3 %	3.85 %
**Operative time**	45 min	50 min
**Hospital stay**	3.5 days	4 days

## Conclusion

LC is the gold-standard for the elderly too. Results are quite similar in both groups thanks to an accurate selection of cases.
